# Cancer therapy–related salivary dysfunction

**DOI:** 10.1172/JCI182661

**Published:** 2024-09-03

**Authors:** Cristina Paz, Annemarie Glassey, Abigail Frick, Sarah Sattar, Nicholas G. Zaorsky, Grace C. Blitzer, Randall J. Kimple

**Affiliations:** 1 Department of Human Oncology, University of Wisconsin School of Medicine and Public Health, Madison, Wisconsin, USA.; 2 University Hospitals Seidman Cancer Center, Cleveland, Ohio, USA.; 3 Case Western Reserve University, Cleveland, Ohio, USA.; 4 University of Wisconsin Carbone Cancer Center, University of Wisconsin School of Medicine and Public Health, Madison, Wisconsin, USA.

## Abstract

Salivary gland dysfunction is a common side effect of cancer treatments. Salivary function plays key roles in critical daily activities. Consequently, changes in salivary function can profoundly impair quality of life for cancer patients. We discuss salivary gland anatomy and physiology to understand how anticancer therapies such as chemotherapy, bone marrow transplantation, immunotherapy, and radiation therapy impair salivary function. We discuss approaches to quantify xerostomia in the clinic, including the advantages and limitations of validated quality-of-life instruments and approaches to directly measuring salivary function. Current and emerging approaches to treat cancer therapy–induced dry mouth are presented using radiation-induced salivary dysfunction as a model. Limitations of current sialagogues and salivary analogues are presented. Emerging approaches, including cellular and gene therapy and novel pharmacologic approaches, are described.

## Introduction

Xerostomia, the subjective feeling of dry mouth typically accompanied by hyposalivation (1–3), is a common but often overlooked condition that can profoundly impair an individual’s quality of life. Xerostomia mainly presents as decreased production of saliva (hyposalivation) by major salivary glands. However, patients may experience xerostomia with no notable decrease in saliva production, but display changes in saliva composition (sialochemistry dysfunction) (1–3). Xerostomia can be caused by multiple etiologies. It can occur naturally in the elderly (4, 5), as a result of autoimmune disorders (6), as a side effect of some medications (7), after nerve damage to the head and neck (8), or as a consequence of chemotherapy, immunotherapy, or radiation therapy (9–14). Patients with head and neck cancer (HNC) are among the most likely to develop xerostomia (15). Most oncologists, and many physicians, lack comprehensive training in oral health, resulting in a disconnect between patient experiences of xerostomia and provider understanding of the challenges patients endure. An opportunity to improve the multidisciplinary care of many cancer patients exists.

**Figure 1:**
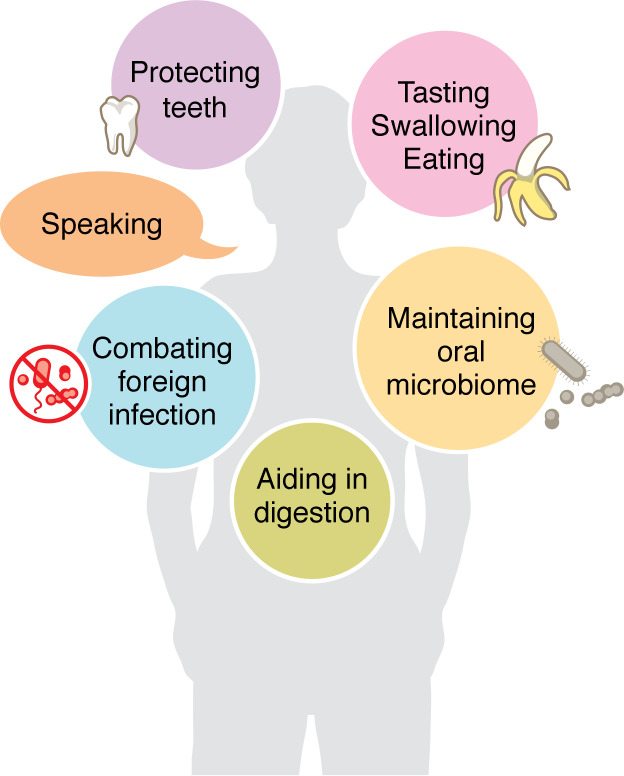
Schematic of the various physiological roles saliva is critical in supporting. Summary of saliva’s contributions to various physiological and behavioral functions. Changes in saliva production or the chemical makeup of saliva as seen in xerostomia can negatively affect all of these aspects of a patient’s life, decreasing overall quality of several facets of the patient’s life.

Saliva is a critical component of healthy oral function and plays an important role in maintaining oral cavity homeostasis. Saliva lubricates the oral mucosa, prevents tooth demineralization, has antimicrobial properties, maintains mouth pH, initiates starch digestion, and is critical for mastication, swallowing, and speech (16, 17). Damage to the major salivary glands leads to a plethora of adverse side effects due to the functions of saliva in normal health (Figure 1). This can include difficulty eating, taste disorders (i.e., dysgeusia), painful tongue, swallowing and chewing difficulties, increased dental caries, speech impairments, pain or burning in the mouth, ulcers of the oral mucosa and tongue, and increased susceptibility to oral infections (10, 16, 18). These effects substantially impact the quality of life of patients with xerostomia (19). The current treatment strategy for xerostomia is primarily palliative and there are few effective long-term treatments. Understanding the pathophysiology underlying xerostomia caused by cancer treatments is critical to developing better treatments that address the root causes of xerostomia rather than just addressing symptoms.

In this Review, we aim to briefly describe salivary gland anatomy and physiology and to summarize the currently available research related to the pathophysiology of xerostomia in cancer patients. The majority of studies investigating the pathophysiology of cancer therapy–induced xerostomia focus on the effects of radiation. Thus, we will use radiation-induced xerostomia (RIX) as a model for cancer therapy–related salivary dysfunction, while identifying what is known about how other cancer therapies impact salivary tissue. Finally, we will address the currently published preclinical and clinical studies related to developing novel therapies for xerostomia. Current treatments for xerostomia such as sialagogues or salivary analogues (see reviews by Jasmer et al., ref. 20; Kapourani et al., ref. 21; and Spirk et al., ref. 22) are briefly discussed.

## Salivary gland anatomy and physiology

**Figure 2:**
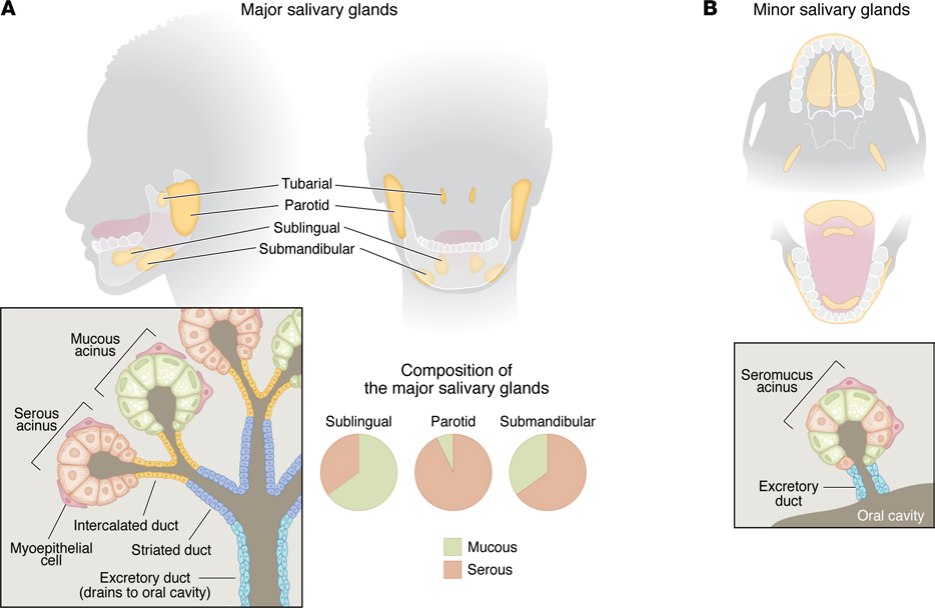
Salivary gland anatomy. (**A**) Location of major salivary glands (yellow) in the human are displayed in two projections. The lower left inset shows the general glandular structure, depicting serous acini, mucinous acini, myoepithelial cells, and ducts. To the right of the inset, pie charts convey differences in the mucous and serous composition of the major salivary glands. (**B**) Minor salivary glands are widely dispersed throughout the oral cavity, as indicated by yellow regions. The inset represents the structure of minor salivary glands, which consist of single seromucinous acini draining directly into excretory ducts.

In humans there are three pairs of major salivary glands — sublingual, submandibular, and parotid — that secrete saliva through a system of salivary ducts (Figure 2). An additional salivary gland, the tubarial gland that lies within the nasopharynx, has recently been described; while tubarial glands share histology with salivary glands, their physiologic function remains uncertain (23–25). The lining of the mouth and throat is also populated by hundreds of minor salivary glands that are located submucosally and secrete saliva directly into the mouth/throat.

Saliva is a complex biofluid that is composed of 99% water and 1% other components such as ions, proteins such as enzymes and growth factors, microorganisms, and some immune cells. Acini are the secretory units of the salivary gland. Serous acini produce a watery, serous saliva that is rich in proteins such as amylase that provides enzymatic activity important to starch digestion (26, 27). Mucinous acini produce a viscoelastic solution rich in mucins that plays an important role in lubrication and hydration of surfaces (28–30). The relative abundance of serous versus mucinous acini dictates the type of saliva produced (i.e., serous, mucinous, or seromucinous) (31). Among the three major salivary glands, the parotid gland produces more serous saliva, the sublingual gland produces more mucinous saliva, and the submandibular gland produces a mixed seromucinous solution. The type of saliva produced is dependent on the relative abundance of the different acinar cells present (32, 33).

In addition to the major salivary glands, minor salivary glands are critical to maintaining basal moisture within the oral mucosa. Most minor salivary glands are mucinous. A small area near the circumvallate papillae contains minor salivary glands producing serous saliva called von Ebner glands, which are believed to be critical to taste perception (31, 34).

Saliva produced in the acini is carried to the oral cavity via a series of ducts. The saliva produced in the acinar cells initially enters the acinar lumen. This lumen is continuous with the lumen of the intercalated ducts, which house cells secreting lysozyme and lactoferrin, proteins with bacteriolytic activity that play an important role in defense against pathogens (26, 35–38). The saliva solution empties from the intercalated ducts into the striated ducts, which are responsible for salt reabsorption and secretion of electrolytes into saliva. The striated duct cells are impermeable to water and reabsorb sodium and chloride and secrete potassium and bicarbonate, making the saliva hypotonic. Striated cells also secrete kallikrein (important for leukocyte attraction) and epidermal growth factor (39, 33). After leaving the striated duct, the salivary solution travels into interlobular/excretory ducts, which terminate in the oral cavity (31, 33). The final saliva solution that enters the oral cavity is hypotonic, which allows for the tasting of salts in food (31).

Salivary secretion is regulated by the autonomic nervous system, with parasympathetic and sympathetic nerves that supply acinar and ductal cells and a large supply of arterioles surrounding the ducts and acini (16, 31, 33, 32, 40). Saliva secretion rates vary according to the level of stimulation, time of day, sex of the organism, and environmental temperature (31, 33, 41). In the first stage of saliva formation, ribosomal proteins in acinar cells synthesize salivary proteins, such as amylase and mucins, which are then packaged and released into the lumen via exocytosis or vesicular transport (33). Sympathetic neurons regulate protein secretion and parasympathetic innervation regulates the transport of water in the salivary gland via muscarinic signaling (M1 and M3 receptors) (31, 42, 43). The final concentration of saliva is regulated by the transport of ions through the activity of the sodium/potassium ATPase and chloride channels (TREM16A) in the apical membrane of acinar cells. The movement of salt into the acinar lumen leads to the movement of water via osmosis through aquaporin channels, namely aquaporin 5 (AQP5) in the apical membrane (31). The primary saliva solution secreted from the acinar cells is a pH-neutral isotonic fluid. The pH has been shown to decrease both during and two years after radiation treatment (44, 45).

Saliva also plays a role in maintaining a healthy oral microbiome (46). Head and neck radiation has been linked to an increased abundance of *Lactobacillus* and a decrease in the overall α diversity of the microbiome up to five years after therapy (47–50). These findings suggest that radiotherapy may result in chronic microbiome changes in patients and highlights the importance of considering microbiome changes in future studies examining the late toxicity of cancer therapy.

## Quantification of xerostomia in the clinic

There are two primary ways to quantify salivary dysfunction in patients: patient-reported outcomes and direct measurement of salivary production.

Several validated quality-of-life (QoL) instruments are used to assess xerostomia. The three most common QoL questionnaires include the University of Michigan Xerostomia-Related Quality of Life Scale (XeQOLS), the Visual Analogue Scale (VAS), and the MD Anderson Dysphagia Inventory (MDADI) (51, 52). These scales are designed to detect QoL changes in xerostomia and associated symptoms, including dysphagia, in patients undergoing active treatment. An important limitation of QoL instruments is that while they are sensitive to acute changes in salivary function, as patients adapt to their “new normal,” they can lose sensitivity to minor changes in salivary function in the years following radiation (53).

Salivary dysfunction can be quantified by direct measurement of saliva production (reviewed in ref. 54). Radiation often results in a decrease in salivary flow rate, beginning shortly after radiation therapy and continuing for several years (52, 55). The amount of saliva is likely not the only contributing factor to xerostomia, as differences in salivary composition are associated with more severe xerostomia regardless of the amount of saliva produced (28). Evaluating protein content in collected saliva, measuring pH levels of saliva, and assessing the oral microbiome are also good methods to distinguish changes in sialochemistry, providing more information than just rates of saliva production, and are metrics in some studies evaluating saliva from HNC patients after radiation (28, 50, 56, 57). While quantification of timed salivary production is methodologically straightforward, even within healthy control patients test-retest reliability can show significant differences (58).

Imaging is a less common way of quantifying salivary gland dysfunction (59, 60). Several imaging approaches have been used to assess salivary dysfunction but are primarily of investigational interest at this time. Ultrasound imaging, including acoustic radiation force impulse (ARFI) imaging, is an effective method to determine both the size and stiffness of salivary glands (61, 62). ARFI uses acoustic compression to determine the shear wave velocity (SWV) of the gland. SWV is a measurement of stiffness, a surrogate for fibrosis that provides a noninvasive approach to monitor fibrosis in irradiated salivary glands of patients with HNC (62, 63). Salivary gland size can also be determined by cross-sectional imaging such as computed tomography or magnetic resonance imaging (MRI). Diffusion-weighted MRI can be used to quantify cellular changes through computation of apparent diffusion coefficient values (64). MRI may also be useful to differentiate etiologies of xerostomia through assessment of ductal deformities, fat deposition, and sialography (65). Sialoscintigraphy is a noninvasive, objective approach to evaluate salivary dysfunction. This method is a nuclear diagnostic imaging technique that uses technetium-99m pertechnetate (^99m^TcO_4_^–^) to measure uptake and excretion in the salivary gland (66, 67). Most recently, prostate-specific membrane antigen (PSMA) PET scans have been used to identify secretory cell loss in salivary glands (68), but how this correlates with salivary function remains unclear.

Finally, mucosal biopsies and other functional tests are used to evaluate xerostomia in other clinical contexts, like Sjogren syndrome (69), but are not part of the clinical recommendations to evaluate xerostomia in cancer patients (70). A “wafer test” has been used as a screening test for identifying xerostomia in patients with connective tissue disease (71), but has not been used in cancer patients. Together, these methods allow clinicians and researchers to monitor the development of xerostomia in patients and determine strategies to provide relief to their patients.

## Effects of cancer therapy on the salivary glands

### Chemotherapy.

Most research on salivary dysfunction in cancer patients is focused on patients with HNC. However, many cancer patients receiving chemotherapy for cancers outside the head and neck region also experience oral sequelae, including mucositis, xerostomia, and dysgeusia. In a prospective cross-sectional study of 155 patients, those receiving chemotherapeutic regimens, including fluorouracil (5-FU), reported xerostomia in 56%–69% of cases, while those receiving taxanes reported xerostomia in 56% of cases (72). Overall, data on effects of chemotherapy, including the duration of changes in salivary function, is lacking, particularly in randomized studies (Table 1). Heterogeneity in data collection and the numerous types of chemotherapy involved make broad statements challenging. Some published studies (reviewed in refs. 9, 73) suggest decreased saliva production and xerostomia in patients during and after chemotherapy for adult and childhood cancers (74, 75), while others have not identified differences (76, 77). In pediatric patients, oral sequelae (not precisely defined, but including xerostomia) is seen in over 50% of patients, with a high incidence in those who received myeloablative therapy prior to bone marrow transplantation (78, 79). This is comparable to the prevalence of xerostomia reported in adults undergoing bone marrow transplantation (reviewed in ref. 9). Patients receiving high-dose radioactive iodine treatment develop xerostomia in approximately 33% of cases (reviewed in ref. 9). Unfortunately, many trials evaluating the efficacy and safety of chemotherapeutic regimens do not include xerostomia in their measurements of adverse effects (Table 1). The lack of data collection is a considerable limitation to understanding the true impact of these therapies on xerostomia. In addition, most studies of xerostomia in patients receiving chemotherapy report data on its incidence only during active therapy, with no long-term data available (72, 73, 78, 79).

### Immunotherapy.

Immune checkpoint inhibitors can induce persistent immune-related adverse events. Between 5% and 20% of patients treated with immune checkpoint inhibitors are reported to have developed xerostomia (80, 11). Xerostomia related to checkpoint inhibition can become a chronic issue impacting quality of life, but often does not start until 3–4 months after the initiation of therapy (81). In one cross-sectional study, xerostomia was seen in 7.4% of patients up to one year and 8.6% of patients more than one year after discontinuation of immune checkpoint inhibitor therapy (82). Lymphocytic infiltration of CD3^+^ T cells, acinar atrophy, and fibrosis have been described in patients with xerostomia following immune checkpoint therapy, and a response to steroids in these patients suggests that inflammation may also play a role (81, 83). Better reporting of oral adverse events from immunotherapy to quantify the incidence and severity as well as additional work to understand the mechanisms underlying immunotherapy related salivary dysfunction are needed.

### Radiotherapy.

External beam radiation to the salivary glands results in a decrease in salivary flow rate and differences in salivary composition, both shortly after radiation and several years later (52, 55, 84). In addition to the radiation dose to the major salivary glands, the presence of minor salivary glands throughout the oral cavity explains the importance of the radiation dose received by the oral cavity in the development of radiation-induced xerostomia (85, 86). Both mucin and amylase decrease after radiation (35, 52). While total protein is associated with an inflammatory state and has been found to increase transiently after radiation, it tends to return to normal ranges in 2 years (44, 45, 87). Salivary pH decreases both during and after radiation treatment (44, 45). Although the short-term analysis of saliva during and after radiation has been investigated, few studies have investigated the late effects of radiation therapy in patients with HNC, with limited studies investigating salivary characteristics five or more years after radiation therapy (88).

Advances in the technical delivery of radiation therapy have greatly improved the ability of a radiation oncologist to specifically target areas of interest while avoiding or minimizing radiation exposure to normal tissues such as the salivary glands. However, due to the anatomy of the head and neck region, the proximity of tumors to salivary tissues makes it challenging to fully avoid delivering radiation to all saliva-producing tissues. With older radiation techniques, 50%–70% of patients receiving radiation therapy to the head and neck developed xerostomia (89, 90). Modern radiation delivery can decrease the incidence to around 30%, depending on the disease stage (91, 92). Recovery of salivary function after radiation depends largely on the dose of radiation delivered to the salivary glands (93, 94). In the best cases, salivary impairment resulting from radiation therapy will only persist for a few months after treatment. However, in many cases this condition is irreversible and patients remain in need of an effective solution. There is a critical need for studies investigating long-term effects of modern radiation.

PSMA-targeted radionuclide therapy can also induce xerostomia due to high expression of PSMA on salivary tissue. Estimates of xerostomia incidence in patients treated with [^225^Ac]AC-PSMA-617 range from 30% to 75% (95–97). In several studies, xerostomia was the dose-limiting toxicity (98). This is similar to data reported from [^177^Lu]LU-PSMA therapy, in which the incidence and severity of xerostomia becomes more common with repetitive dosing (99–101).

Patients with RIX demonstrate sialochemistry dysfunction and hyposalivation (20, 93). The pathophysiology of RIX includes acinar cell atrophy and chronic inflammation within the salivary glands. These result in alterations in salivary volume, pH, and viscoelasticity (i.e., stickiness) (90). Acinar cells have a slow turnover rate and are highly differentiated, suggesting that they would be resistant to the effects of radiation, but based on early changes in salivary composition and flow, salivary glands are in fact functionally radiosensitive (102). Initial theories of this loss of function were attributed to apoptosis, lysis, or leakage of granules (103); however, there is no evidence supporting these theories.

**Figure 3:**
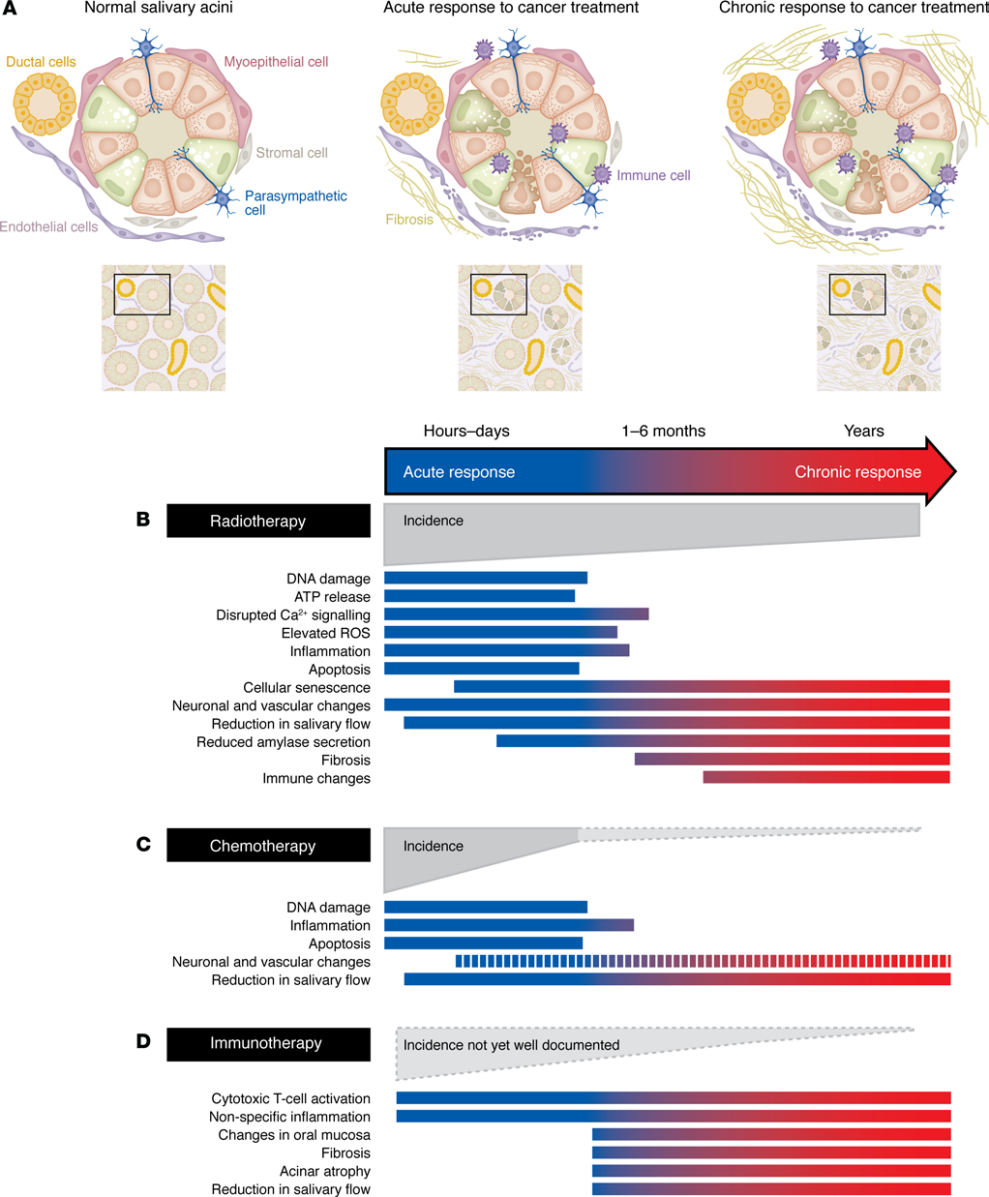
Effects of cancer therapies on the salivary glands. (**A**) Acute and chronic responses to cancer treatment are associated with fibrosis and damage to salivary acini, including changes to endothelial and myoepithelial cells as well as serous and mucous epithelial cells. Recent studies have indicated a role for T cell activation and other indicated changes in immunotherapy-related salivary dysfunction, while macrophages have been shown to be involved in damage response following radiotherapy. (**B**) Timeline of known effects of radiotherapy, chemotherapy, and immunotherapy on salivary glands. Solid bars represent approximate start and end time of indicated changes; a dashed bar indicates presumed changes. Gray wedge indicates the decline of overall incidence of these responses in patients over time. Effects of radiotherapy have been well described; however, few descriptions of mechanisms underlying chemotherapy or immunotherapy-driven changes have been described. Adapted from Jasmer et al. (20).

Salivary gland dysfunction because of radiation exposure typically occurs in two phases, acute and chronic (Figure 3). For some patients, xerostomia resolves after the acute phase. However, many patients suffer from chronic xerostomia and continue to experience symptoms well after therapy ends. Early changes in salivary function during radiation treatment are attributed to damage to the plasma membrane of secretory cells. This damage interferes with water secretion and is responsible for the relatively rapid thickening of saliva experienced by patients. In many, but not all patients, almost immediately after radiation exposure (hours to days) there is a 50%–60% decrease in the amount of saliva produced, a loss of acinar cells, shrinkage of the gland, and changes in the composition of saliva (104, 105, 20). Saliva from damaged glands has higher concentrations of mucins, a change in pH from neutral to acidic, altered viscoelasticity, and increased osmolarity (106). Chronically affected individuals continue to have significant decreases in saliva volume, changes in salivary composition that can lead to mucositis, and sustained reductions in the size of the acinar compartments due to death of acinar stem cells (20, 105, 107). The surrounding environmental damage evidenced by fibrosis and inflammation produces an environment that is suboptimal for proper function (107). Parasympathetic and sympathetic innervation of salivary glands are also impaired by radiation exposure and can contribute to reduced functionality of salivary glands (42, 43, 108, 31). Parasympathetic signaling can support the regeneration of salivary gland tissue. In mice, administration of neurturin, a neurotrophic factor that reduces parasympathetic nerve apoptosis, led to improved regeneration of salivary epithelial tissues (109). Finally, although not well described, some groups have reported changes in the immune landscape of the gland (20, 105, 107, 110).

## Treatment of xerostomia

Current treatments for xerostomia focus on either prevention of xerostomia through reducing damage to the salivary glands or increasing the volume of functional saliva using manufactured substitutes. Modern radiation techniques such as intensity-modulated radiation therapy (IMRT) deliver lower radiation doses to the glands than older techniques and result in lower rates of xerostomia (111). Approaches to move the salivary glands out of the radiation field have also been used in patients with HNC (112–117). Sialagogues (i.e., drugs that increase the flow of saliva) and salivary analogues can provide temporary relief for patients, but do not change the underlying glandular dysfunction. Additional approaches such as acupuncture and hyperbaric oxygen treatments have limited data supporting their efficacy (118, 119). Current strategies to prevent or treat salivary dysfunction are summarized in Table 2.

## Emerging approaches to treat xerostomia

### Gene therapy.

Many groups are investigating the utility of gene therapy in the treatment of RIX. These studies focus on increasing expression of water channels in salivary cells to facilitate fluid transport and hopefully increase saliva production. The water channel gene *AQP1* (aquaporin 1) encodes a constitutively active water channel that facilitates fluid secretion along an osmotic gradient. AQP1 is expressed in the myoepithelial and endothelial cells of humans and mouse salivary glands and is limited to endothelial cells in rat submandibular gland (SMG) (120). This section addresses both in vitro and in vivo studies that aim to increase expression of AQP1.

An in vitro study explored artificial induction of AQP1 expression in established salivary gland cell lines and primary human salivary progenitor cells via two different methods: artificial transcriptional complexes and epigenetic alterations. Wang et al. introduced an artificial transcriptional complex at the *AQP1* gene and saw increased expression of AQP1 in cell lines after delivery of guide RNAs targeting the promoter region (121). This group also explored the impact of epigenetic modification on AQP1 expression, in which they performed chemical demethylation of A253 cells (derived from human SMG tumor) and saw increased expression of AQP1 through demethylation alone (121). These data support other literature that suggested methylation was the primary method for *AQP1* gene silencing (122). This in vitro study suggests that epigenetic editing can hold potential for inducing AQP1 expression in salivary cells.

The adenoviral delivery of human *AQP1* (AdhAQP1) utilizes an adenovirus-derived vector to deliver the *AQP1* gene into infected salivary cells. Many groups use this method to investigate *AQP1* gene delivery in in vitro and in vivo models. One study observed a two- to three-fold increase in salivary fluid secretion compared with control animals after AdAQP1 delivery to rat SMG 3–4 months after radiation (17.5 or 21 Gy in a single fraction) (123). Another group delivered AdhAQP1 to the minipig parotid gland and showed improved saliva secretory volume, but not changes in salivary composition, within 8 weeks after administration (124). The minipig is a highly translational model animal, useful for evaluating novel therapies to prevent or reverse RIX in humans. AdhAQP1 has shown clinical promise in a phase I clinical trial in patients with RIX (ClinicalTrials.gov NCT00372320). Researchers evaluating late responses to the therapy found that AdhAQP1 resulted in both short- and long-term improvement of parotid salivary flow and sustained symptomatic relief for 2–3 years (125). This approach is being further developed in a recently completed phase I study (ClinicalTrials.gov NCT02446249) and an ongoing randomized double-blind placebo-controlled study (ClinicalTrials.gov NCT05926765).

### Stem cell therapy.

There is substantial interest in modulating salivary gland stem cells to improve salivary function. Studies in mice and humans have demonstrated that there are regions with high stem cell density within the parotid gland, and damage to these regions is a good predictor of salivary dysfunction after radiation (126). A double-blind randomized controlled trial was performed to determine the impact of dose reduction in these regions on salivary flow from the parotid gland. Salivary flow was reduced 16.8% and 8.5%, and patient-reported xerostomia was 50.0% and 45.9% in the standard IMRT versus high stem cell density–sparing IMRT groups, respectively. Unfortunately, in this trial, salivary flow and perceived xerostomia were not significantly improved (127).

Salivary stem cell populations are only beginning to be defined in recent years, as prior to this there was not a consensus definition of what constitutes the salivary stem cell population. Researchers identified a population of SOX2^+^ adult human salivary gland progenitor cells in the three major salivary glands that could potentially differentiate into acinar cells (128). Emmerson et al. demonstrated that SOX2 was essential for salivary gland regeneration following a single dose of 10 Gy to the murine sublingual gland (128). Using an ex vivo model, SOX2^+^ cells could repopulate the irradiated murine sublingual gland. It is possible that the presence of senescent cells, as a consequence of radiation, can enhance the self-renewal potential of the remaining, nonsenescent salivary gland stem cells, like these SOX2^+^ progenitor cells (126). Several groups have also been utilizing induced pluripotent stem cells to establish salivary tissue for in vitro and in vivo modeling (129–131). These models appear to recapitulate the stem cell populations seen in the developing gland and may serve as important models for future translational research.

There are two populations of stem cells identified in the ductal regions of the gland that have demonstrated the ability to regenerate ductal tissue after radiation exposure; these populations are marked by cytokeratin 14 (KRT14) and Kit, respectively (132, 133). Both KRT14^+^ and Kit^+^ cells demonstrated the ability to regenerate salivary tissue through distinct mechanisms. KRT14^+^ cells are fast-cycling cells that maintain a K14^+^ cell population in granulated ducts under homeostasis and also expand in response to radiation or severe injury and divide to produce cells in the larger granulated ducts, Aqp5^+^ acinar cells, and Kit^+^ intercalated duct cells (132–135). KRT14^+^ cells are now considered to be a bona fide salivary stem cell marker, as researchers have demonstrated the capacity of KRT14^+^ cells to replenish injured glands on many occasions; however, most studies show that KRT14^+^ cells replenish cells in the ductal compartment and do not contribute to acinar regeneration (132, 134–136).

Early work in identifying these salivary stem cell types used 3D salispheres generated from murine SMGs to study the expansion of potential salivary gland stem cells. Lombaert et al. generated salispheres from murine SMG tissue, and within these populations they identified cells expressing the stem cell markers Sca-1, Kit, and Musashi-1 (137). They demonstrated histologically that the isolated spheres initially expressed mostly ductal marker and were eventually capable of producing mucins and amylase. These data indicate that the spheres they isolated were of ductal origin, and with time, differentiated into cells with an acinar phenotype. After characterization, the spheroids were transplanted into irradiated murine salivary glands and the group saw improvement in glandular structure and increased proliferation compared with controls. They used fluorescence-activatedcell sorting (FACS) to enrich for Kit^+^ stem cells to further characterize the salivary stem cells present in the spheroid cultures. Kit^+^ cells were then transplanted into the salivary glands of irradiated female mice, while controls received Kit^–^ populations. After 90 days, transplantation of Kit^+^ cells resulted in glands showing a similar morphology to nonirradiated glands, including restored acinar cell populations and increased saliva production in 69% of animals (137). The Kit^–^ transplantation only resulted in minor responses. This led researchers to investigate whether this Kit^+^ population of spheroids are responsible for acinar regeneration in damaged tissues. Nanduri et al. isolated Kit^+^ spheroids from mice and injected them into irradiated murine salivary glands, and this resulted in improved gland architecture and improved saliva production compared with controls. They continued to study these Kit^+^ spheres and the impact of spheres coexpressing other salivary stem cell markers like CD24 and CD49f on irradiated salivary tissues, with positive results (138, 139). This approach has also been used to isolate human Kit^+^ cells from salivary tissue and implant them in murine salivary glands; results demonstrated restoration of salivary gland function after irradiation in a xenotransplantation approach (140).

However, the identification of a Kit^+^ stem cell in the salivary gland has been disputed by other studies, including those from the Ghazizadeh group (135, 136). While this group also identified distinct Kit^+^ and KRT14^+^ cells in the salivary gland, they used lineage tracing studies to show that the KRT14^+^ population is the major source of regeneration (132, 135). A different study by Nanduri et al. also discovered that whether or not a spheroid is Kit^+^ is not critical to their effectiveness at regenerating tissue. They identified a population of CD24^hi^CD29^hi^ spheroids that produced the best regenerative response to radiation regardless of Kit positivity, indicating this Kit^+^ cell population does not further enrich for stem cells (141). Kwak et al. concluded that Kit is not a reliable marker for salivary stem cells, and suggest that a better marker when considering clinical implications is KRT14 (136). Ninche et al. also identified KRT14^+^ cells as a reliable salivary stem cell marker (135). Interestingly, in their study, they demonstrated that acinar regeneration relies on methods independent of KRT14^+^ ductal stem cells. Their group found that in severe injury (ligation) models, KRT14^+^ cells contribute to generation of granular ductal cells, Kit^+^ intercalated duct cells, and Aqp5^+^ acinar cells. Using lineage tracing experiments, they showed that most of the acinar replenishment in the severe injury model is contributed by dedifferentiated KRT14^+^SMA^+^ myoepithelial cells. Upon injury, the myoepithelial cells transdifferentiate into a bipotent progenitor state capable of producing acinar cells and Kit^+^ intercalated duct cells. They also found that Kit^+^ intercalated duct cells are capable of transdifferentiation into a bipotent progenitor cell type as well, capable of producing both acinar cells and more Kit^+^ intercalated duct cells. These data indicate that Kit^+^ cells in the intercalated duct can act as a reservoir of acinar progenitors in the case of injury, but are not the main contributor (135). Despite the confounding opinions of what is and is not a salivary stem cell, these data suggest that the salivary gland maintains mechanisms capable of regeneration. Use of salivary organoids, and specifically stem cell–derived three-dimensional models, is being studied in an ongoing clinical trial, as an autologous source of transplantable material by a group in the Netherlands (140, 142).

We, and others, have utilized mesenchymal stromal cells (MSCs) to improve salivary function after radiation (143–146). MSCs are most commonly isolated from adipose tissue or bone marrow. When injected into the irradiated salivary glands of mice, they improve salivary function (147–149). This approach is being tested in ongoing clinical studies run by several groups (ClinicalTrials.gov NCT04489732, NCT04776538, NCT03876197, NCT03874572, and NCT03743155). The MESRIX study conducted by Rigshospitalet in Denmark looked at the effects of injecting autologous adipose-derived MSCs, or MSC(A), into the SMGs of patients. In this blinded randomized controlled trial, HNC patients with xerostomia had MSC(A) isolated, expanded, and injected into the submandibular gland. The delivery of MSC(A) was shown to be safe and demonstrated a significant increase in salivary flow at one and four months. Compared with baseline, symptoms of xerostomia were significantly reduced, and there was increased serous tissue in the glands (143, 145, 150). The team conducting the MESRIX study then completed a trial investigating the safety of allogeneic MSC(A) (151), which led to their opening a randomized phase II study (MESRIX-III, ClinicalTrials.gov NCT04776538) in order to evaluate the safety and efficacy of injection of MSC(A) from healthy donors (allogeneic) in patients with HNC suffering from xerostomia (152). A multidisciplinary team at the University of Wisconsin recently published a pilot study demonstrating the safety of autologous marrow–derived MSCs, or MSC(M), in patients who were two or more years out from the completion of radiation or chemoradiation (146). They recently opened a phase I study (ClinicalTrials.gov NCT05820711) utilizing autologous MSC(M) to define a phase II dose of cells (153). The optimal source of MSCs (marrow, adipose, or other; allogeneic or autologous) remains to be defined.

An adjunct to injecting stem cell populations into damaged glands is to introduce biomaterials to support regeneration of damaged glands. Several labs are testing novel biomaterial approaches to engineer implantable tissue that exhibit the regenerative potential of isolated stem or progenitor cells (154). Others hope to regenerate salivary glands by using primary murine SMG cells to build cell sheets to repair damaged regions of the tissue (155). These stem cell–based therapies show preclinical and clinical promise and may represent the next major step in the treatment or prevention of RIX.

### Pharmacological intervention.

In addition to gene therapy and stem cell therapy methods, many researchers are exploring pharmacological approaches to treat or prevent RIX. Minipigs receiving an injection of rapamycin, an inhibitor of mTOR signaling, one hour prior to radiotherapy had improved saliva flow rates 12 weeks following treatment (156). Another potential pharmacological intervention for salivary gland regeneration is the postirradiation delivery of ectodysplasin A receptor (EDAR)-agonist monoclonal antibodies. EDAR is a signaling molecule involved in salivary gland development. Transient activation of EDAR signaling after ionizing radiation (5 Gy) restored salivary gland function and amylase levels after 90 days in mice (157).

Regenerative treatments to restore salivary gland functions after radiation therapy require further investigation, but provide a promising outlook for relief and prevention of RIX symptoms.

## Conclusions

Xerostomia is a serious cancer treatment–related toxicity that has long-term consequences for survivor’s quality of life. It represents a major unmet medical need given the poor efficacy of available treatments. The number of open and unreported clinical trials may suggest that investigated therapies have largely failed to produce beneficial results. Advances in the understanding of the molecular and immunologic mechanisms underlying the development of xerostomia following radiation and other cancer treatments may identify new approaches and therapeutic opportunities. Novel trials of cellular therapies in the last few years are beginning to demonstrate encouraging results (145, 146, 151), supporting the need for further investigation into these therapies. Continued investment in basic research, translational studies, and clinical trials is needed to help grow the field of salivary biology.

Patients with cancer therapy–induced xerostomia should have close follow-up with an oral health provider such as a dentist. More frequent oral health surveillance and implementation of oral hygiene strategies can be used to maintain a healthy mouth. Early management of dental caries and management or prevention of opportunistic oral infections can improve overall quality of life for patients after a cancer diagnosis (158).

## Figures and Tables

**Table 2:**
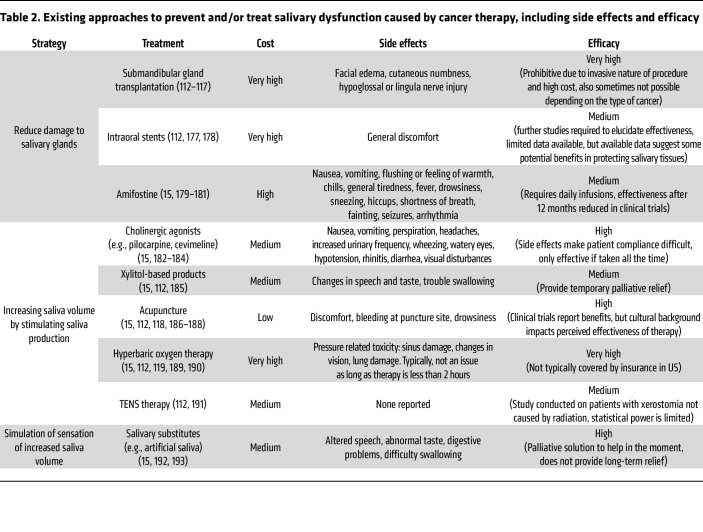
Existing approaches to prevent and/or treat salivary dysfunction caused by cancer therapy, including side effects and efficacy

**Table 1:**
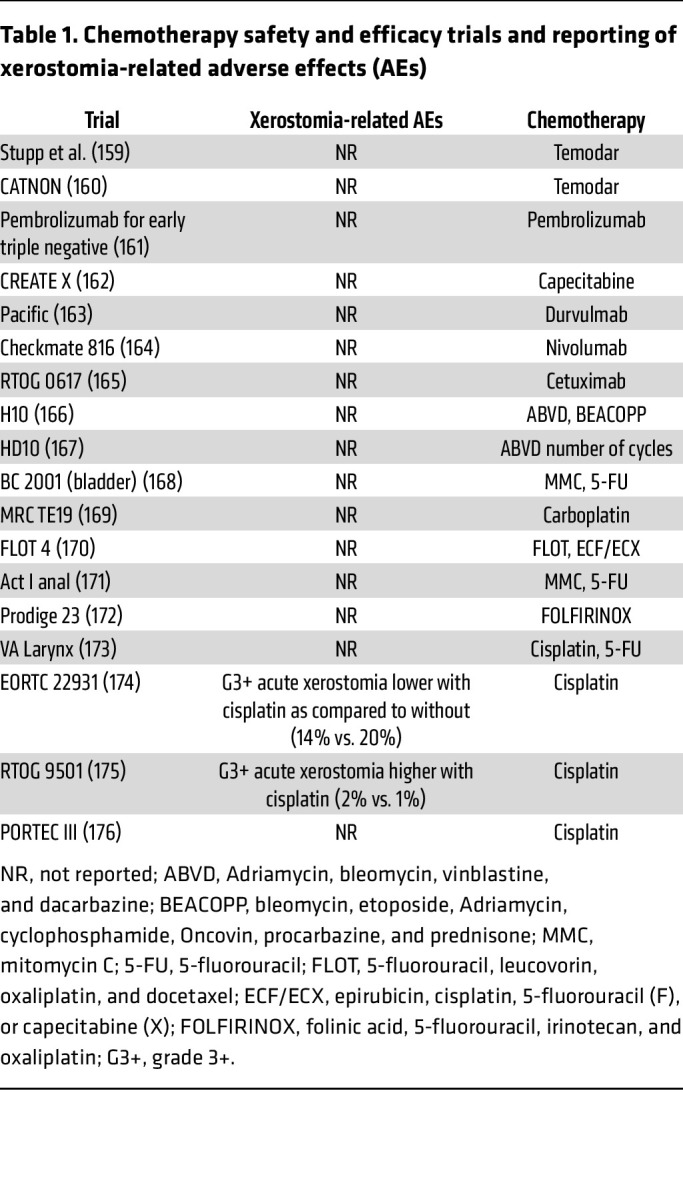
Chemotherapy safety and efficacy trials and reporting of xerostomia-related adverse effects (AEs)

## Version 1 09/03/2024

Electronic publication
